# Differentiation in Rheumatic Diseases (so Called)

**Published:** 1891-03

**Authors:** 


*Differentiation in Rheumatic Diseases (so called).* By HUGH LANE. London: J. & A. CHURCHILL. 1890.

This paper, read before the Bristol Medico-Chirurgical Society, endeavours to analyse and differentiate the various conditions commonly spoken of as chronic rheumatism. The author emphasises the distinction between rheumatoid arthritis proper and the various joint deformities resulting from rheumatism, but nevertheless is obliged to admit the existence of "mixed cases" of rheumatoid and rheumatic arthritis. The mineral waters of Bath, and massage, appear to do more for such maladies than is often believed.

